# Wetlands for Wellbeing: Piloting a Nature-Based Health Intervention for the Management of Anxiety and Depression

**DOI:** 10.3390/ijerph16224413

**Published:** 2019-11-11

**Authors:** Phoebe R. Maund, Katherine N. Irvine, Jonathan Reeves, Emily Strong, Ruth Cromie, Martin Dallimer, Zoe G. Davies

**Affiliations:** 1 The Wildfowl & Wetlands Trust, Slimbridge, Gloucester GL2 7BT, UK; jonathan.reeves@wwt.org.uk (J.R.); emily.strong@wwt.org.uk (E.S.); ruth.cromie@wwt.org.uk (R.C.); 2 Durrell Institute of Conservation and Ecology (DICE), School of Anthropology and Conservation, University of Kent, Canterbury CT2 8NR, UK; z.g.davies@kent.ac.uk; 3 Social, Economic and Geographic Sciences, James Hutton Institute, Craigiebuckler, Aberdeen AB15 8QH, UK; Kate.Irvine@hutton.ac.uk; 4 Sustainability Research Institute, School of Earth and Environment, University of Leeds, Leeds LS9 2JT, UK; M.Dallimer@leeds.ac.uk

**Keywords:** attention restoration theory, biodiversity, blue space, ecosystem services, green space, mental health, mixed methods, nature-based therapy, social prescribing, stress

## Abstract

Nature-based health interventions (NBIs) for the treatment of poor mental health are becoming increasingly common, yet evidence to support their effectiveness is lacking. We conduct a pilot study of a six-week intervention, aiming to engage individuals with wetland nature for the treatment of anxiety and/or depression. We employed a mixed methods design, using questionnaires, focus groups and semi-structured interviews to evaluate the intervention from the perspective of participants (n = 16) and healthcare professionals (n = 2). Results demonstrate significant improvements in mental health across a range of indicators, including mental wellbeing (Warwick and Edinburgh Mental Wellbeing Scale), anxiety (Generalised Anxiety Disorder-7), stress (Perceived Stress Scale) and emotional wellbeing (Positive and Negative Affect Schedule). Participants and healthcare professionals cited additional outcomes including improved physical health and reduced social isolation. The wetland site provided a sense of escape from participants’ everyday environments, facilitating relaxation and reductions in stress. Wetland staff knowledge of the natural world, transportation and group organisation also played a considerable role in the intervention’s success. These aspects should be considered in future and existing NBIs to maximise benefits to participants. We propose NBIs based in wetlands are an effective therapy option for individuals diagnosed with anxiety and/or depression.

## 1. Introduction

The World Health Organisation (WHO) has identified the improvement of mental health as a global priority [1]. In 2021, non-communicable diseases (NCDs), including poor mental health, will surpass all other health conditions as the leading cause of death worldwide [2]. Indeed, mental illness represents the single largest cause of disability and frequently co-occurs with other NCDs due to shared risk factors, including increasing age, low household income, high tobacco and alcohol use, poor diet and physical inactivity [1]. The associated economic burden is significant. For instance, in the UK, mental illness alone accounts for 23% of National Health Service spending, and a total of £34 billion each year across government departments [3]. There are also substantial wider economic impacts, with poor mental health predicted to result in an estimated loss of $16 trillion to the global economy between 2010–2030, primarily due to declines in productivity [4]. Consequently, identifying low-cost and effective mental health treatment options is essential to reduce the pressure on healthcare resources.

An expanding body of evidence suggests that nature plays a role in relieving the symptoms of individuals experiencing poor mental health. Green space has been associated with reductions in stress [5,6,7,8], improvements in cognitive functioning [6,9,10], increases in self-esteem [11] and reductions of depression and anxiety [12,13]. Blue spaces, defined as environments that predominately consist of water, have been shown to be better at promoting wellbeing than green space, across a broad range of mental health indicators [14,15,16,17]. For example, blue spaces are associated with lower levels of anxiety and mood disorders, and positively associated with self-reported mental and general health [18]. However, the vast majority of blue space studies conducted to date have been focussed on coastal environments, with few examining inland freshwater habitats such as wetlands. The work that has been done shows that people living adjacent to urban wetlands benefit from increased positive affect and perceive them as having restorative qualities [19], providing opportunities for ‘being away’ and experiencing ‘fascination’ [20]. Additionally, Reeves et al. [21] found that wetlands may encourage stress recovery, particularly for individuals who are experiencing high levels of self-reported stress. This might be attributable to wetlands’ high levels of biodiversity, as species richness has been shown to be positively associated with self-reported psychological wellbeing [22,23,24,25].

Healthcare professionals and policy-makers are increasingly advocating the use of nature-based health interventions (NBIs), alongside traditional drug and psychological therapies, to manage the growing demands associated with poor mental health [26,27]. NBIs are defined as “*programmes, activities or strategies that aim to engage people in nature-based experiences with the specific goal of achieving improved health and wellbeing”* [28] (pp. 2) through prevention of illness, promotion of general wellbeing or treatment of specific health issues. They can be broadly grouped into two categories: 1) Interventions that alter the environments where people live, work, learn or heal; or 2) interventions designed to change individual behaviour (such as promoting physical activity or engagement with nature) [28]. Despite the recent proliferation of NBIs with an aim to foster a change in an individual’s behaviour, their effectiveness is rarely evaluated [29]. Moreover, the assessments that do exist are principally concentrated on activities taking place in green spaces, such as group walks (e.g., [30,31,32,33]) or gardening, (e.g., [34,35]), with a paucity of evidence about NBIs in blue spaces.

In this study, we investigate the effectiveness of a structured six-week pilot NBI taking place within an inland wetland site, the aim of which was to improve the mental health of individuals experiencing anxiety and/or depression through engagement with nature. Anxiety and depression are both stress-related mental illnesses [36], meaning that the stress-reducing effects of spending time in natural environments may help in treatment of the conditions [18]. Specifically, we examine: 1) What effects the wetland NBI has on the mental health of individuals diagnosed with anxiety and/or depression; and 2) what specific characteristics of the wetland NBI design support nature engagement and wellbeing effect.

## 2. Materials and Methods

### 2.1. Wetland NBI Design

**Figure 1:**
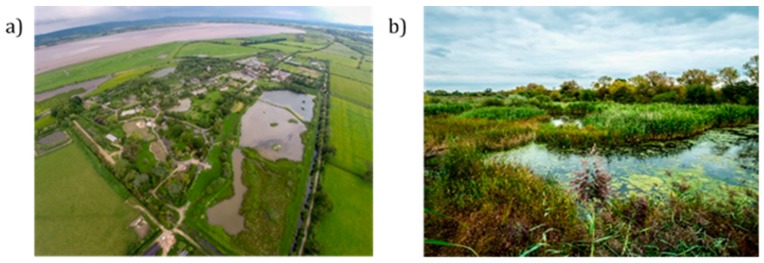
(**a**) The wetland nature-based health intervention took place within a Wildfowl & Wetlands Trust (WWT) wetland site in Gloucestershire, UK. (**b**) The site allows visitors to interact with wetland nature in multiple ways, including canoeing through reed beds. (Photos from WWT).

The wetland NBI was designed to facilitate engagement with nature as a treatment for individuals diagnosed with anxiety and/or depression. Participants took part in a two-hour session per week for six consecutive weeks. The pilot wetland NBI ran twice, once in May 2019 and then repeated in July 2019, with a different set of participants each time. Each session took place within a Wildfowl & Wetlands Trust (WWT) wetland site in Gloucestershire, UK (https://www.wwt.org.uk/wetland-centres/slimbridge/). The site covers 325 hectares, includes permanently flooded areas, and supports high species richness and abundance (Figure 1). Its importance for conservation is recognised through the multiple protected area designations that cover the reserve, including at national level (Site of Special Scientific Interest), European level (a Special Protection Area) and internationally (a Ramsar site).

**Figure 2:**
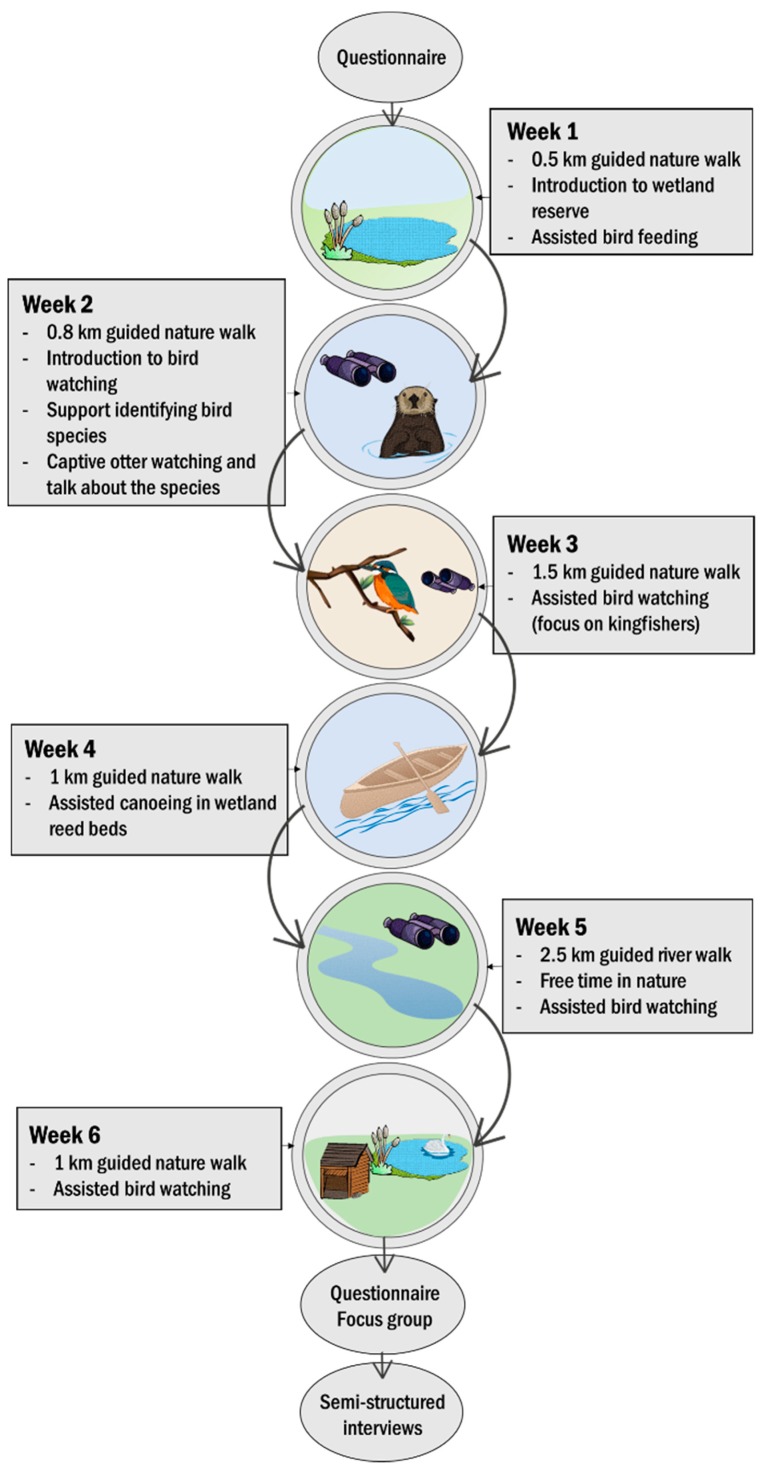
The structured six-week nature-based health intervention (NBI) consisted of a broad range of nature-focused activities that took place within a wetland site. Wetland NBI activities (square boxes) were guided by a minimum of two wetland site staff and one mental health support worker. Data collection activities (circles) included a questionnaire self-reporting on indicators of mental health before and after the six-week intervention, as well as a participant focus group. Interviews were conducted with the two mental health support workers after the wetland NBIs were completed.

On site, there are numerous bird hides that provide visitors with views of charismatic wildlife (e.g., Eurasian cranes, *Grus grus* and kingfishers, *Alcedo atthis*). In addition to bird watching, visitors can take part in a range of activities, such as canoeing and guided walks. The wetland NBI was designed to draw on these opportunities for interaction with nature (Figure 2). Each week participants were transported via minibus to the site from surrounding areas, so they could engage in structured activities, which were guided by a minimum of two wetland site staff members and one trained mental health support worker.

### 2.2. Participant Recruitment

Participants were recruited through a community mental wellbeing service that treats individuals experiencing poor mental health within the local area. This non-governmental organisation offers one-to-one and group therapy, as well as signposting individuals to suitable health promoting interventions delivered by third-party organisations. Typically, activities offered by these third-party organisations take place indoors. For example, clients are commonly referred to art therapy courses or indoor exercise classes. The mental health support workers invited individuals to participate in the wetland NBI who met the following a priori defined inclusion criteria: 1) Aged 18 years or older; 2) be registered with the community mental wellbeing service; 3) diagnosed with depression and/or anxiety (as categorised by mental health support workers); 4) physically able to take part in the range of outdoor activities; and 5) deemed able to provide informed consent. A maximum of 12 participants could participate in each of the two six-week NBIs. All participants provided written informed consent before taking part in the study. Ethical approval was provided by the WWT Human Ethics Committee and the research was conducted in accordance with the Framework for Research Ethics stipulated by the UK Economic and Social Research Council (WWT0118042019).

### 2.3. Questionnaire

Participants self-completed hard copy questionnaires both pre- and post-intervention, collecting data on a variety of commonly used and validated mental health indicators. The pre-intervention questionnaires were conducted at the start of week one, prior to engaging in any wetland NBI activities. The post-intervention questionnaires were undertaken immediately following the final session of the six-week intervention.

Mental wellbeing was assessed by participants rating their level of agreement with statements on the Warwick Edinburgh Mental Wellbeing Scale (WEMWBS) [37]. The 14 items are positively worded (e.g., ‘*I’ve been feeling optimistic about the future’* and ‘*I’ve been feeling loved’*) and refer to participant’s experience over the last two weeks on a 5-point scale (1 = none of the time, 5 = all of the time). Total WEMWBS scores range from 14 to 70, with higher scores representing an increased level of mental wellbeing. In the UK, the National Health Service (NHS) uses scores of 40 or below to define low mental wellbeing, corresponding to probable depression. Scores between 41 and 44 indicate possible depression.

Stress was measured using the Perceived Stress Scale (PSS) [38]. This validated 10-item scale asks participants questions relevant to the last month including ‘*how often have you felt you were on top of things’* and *‘how often have you felt nervous and stressed’*. Answers are on a 5-point scale (0 = never, 4 = very often), with a final total range of 0 to 40, with elevated scores indicating greater psychological stress.

To understand the extent to which participants were experiencing symptoms associated with generalised anxiety disorder (GAD) we used the GAD-7 scale [39]. The 7-item symptom-orientated scale is based on *Diagnostic and Statistical Manual of Mental Disorders, Fourth Edition* (DSM-IV) criteria for GAD diagnosis. Individuals report the frequency they experienced symptoms of anxiety over the past two weeks on a 4-point scale (0 = not at all, 3 = nearly every day; e.g., ‘*how often have you had trouble relaxing’* and ‘*how often have you been worrying too much about different things’*). Total GAD-7 scores range between 0 and 21. Score thresholds of 5, 10 and 15 equate to clinically-graded mild, moderate and severe anxiety respectively.

Positive and negative affect were measured using the Positive and Negative Affect Schedule (PANAS), which effectively comprises two subscales [40]. Participants consider the frequency they experienced both positive (e.g., *‘active’*, *‘inspired’*) and negative (e.g., *‘guilty’*, *‘upset’*) moods over the last two weeks. Responses were measured on a 5-point scale (1 = none of the time, 5 = all of the time), with resulting scores ranging from 10 to 50 for each subscale. Higher scores represented a greater experienced positive or negative affect.

The pre-intervention questionnaire additionally gathered information regarding the sociodemographic background of participants, consisting of gender, age, ethnicity and employment status. Given there was an element of self-selection to engaging in the wetland NBI, these data provided us with an understanding of the types of people who were willing to participate in the intervention.

### 2.4. Focus Groups and Interviews

All participants were invited to take part in a focus group at the end of week six of the wetland NBI after the post-intervention questionnaire was completed. In addition, semi-structured interviews were conducted with the two mental health support workers who were involved in the delivery of the wetland NBI, as well as interacting with the participants outside of the wetland NBI via the community mental wellbeing service one-to-one support sessions. The aim of the focus groups and interviews was to further understand how the participants and mental health support workers described the effects of the intervention, and their interpretation of how the wetland environment and the design of the intervention contributed to the reported outcomes. Interview and focus group guides were developed containing questions focusing on the impacts of the NBI and how the wetland setting and intervention design may have contributed towards those outcomes. Questions included ‘*thinking about your involvement in this programme over the last six weeks, do you think it has had any positive or negative impacts on your mental health*’ and ‘*were there any specific parts of the programme that you believe influenced these impacts*’. Each question was followed by a series of prompt questions (e.g., *‘could you describe some of those impacts further’*) for the interviewer to further stimulate discussion if needed. Interviews and focus groups were conducted in a meeting room at the NBI site. The focus groups and interviews were audiotaped using a digital recorder.

### 2.5. Statistical Analyses

Analyses were undertaken with a combined sample. All statistical analyses were undertaken in R (version 3.5.3, R Foundation for Statistical Computing, Vienna, Austria) [41]. We used a two-sample z-test to elucidate whether the sociodemographic background of the wetland NBI participants was significantly different to the wider community mental wellbeing service membership. Pre- and post-intervention wellbeing indicator scores were compared using Wilcoxon signed rank test for paired data.

The focus groups and interviews were transcribed verbatim and imported into NVivo12 (QSR International (UK) Limited, London, UK). Conventional content analysis [42] was used to assess the described effects of the wetland NBI, in addition to the role of the natural environment and elements of the intervention design that were believed to be responsible for the reported outcomes. This was done both from the perspective of the participants and the mental health support workers. Quotes are used in the results section to illustrate the common key themes that emerged from the analyses.

## 3. Results

### 3.1. Participants

Eighteen individuals were recruited to participate in the wetland NBI (n = 8 for the first NBI, n = 10 for the second). Of these, 16 completed the intervention and were included in subsequent analyses. The two people who withdrew from the wetland NBI reported that they had to leave because of external factors (e.g., stressful life events), rather than the invention itself.

Participants represented male and female genders and were spread across a range of age groups (Table 1). Nearly all of them identified as White British (81%) and the majority were unemployed or retired (63% and 19% respectively). Sociodemographic background generally mirrored the wider community mental wellbeing service membership, although there was a slight overrepresentation of older participants (aged 65-85; z = 6.8, *p* ≤ 0.001) who reported being retired (z = 3.6, *p* ≤ 0.001).

### 3.2. Wetland NBI Mental Health Outcomes

All quantitative measures relating to mental health indicators showed a statistically significant change after the wetland NBI (Table 2). After the NBI, participants had greater mental wellbeing and positive affect, and less anxiety, negative affect and perceived stress. Clinically meaningful differences were observed in WEMWBS scores, with the group mean no longer corresponding to a ‘probable depression’ diagnosis. In the case of our measure for anxiety (GAD-7), pre- and post-intervention scores still aligned with a diagnosis of moderate anxiety. However, there were several cases where changes in individual scores translated to a downgrading of their anxiety (n = 7).

#### 3.2.1. Reducing Symptoms of Anxiety and Depression

In focus groups, participants consistently described a positive relationship between engaging in the wetland NBI and improved mental health. Several participants emphasised how they felt less anxious during the sessions. Exemplar quotes included: “*I feel like it has really helped manage my anxiety”*; “*with anxiety and things it is just there, it is always there. But when you come here, as soon as you are out of the car you never think about this or that or the mental health. It is all about the here and the now*.”

When asked whether there were additional impacts on their mental health, participants spoke about how they were able to more easily relax during the sessions. Comments included: “*You can feel your shoulders just go a bit more relaxed. That tension goes.*” and “*I feel much more relaxed when I leave here each week.*” Similarly, participants reported enhanced positive feelings including happiness and joy, as well as reductions in anger, nervousness and frustration. This was summed up by one participant as: “*Any anger you have just disappears, you can’t think angry thoughts when you have these little things* [birds] *right in front of you.”*

Generally, the mental health support worker perceptions mirrored those of the participants. They explained how they observed participants’ body language alter when they were within the wetland. One mental health support worker described this as a: *“Visible weight being lifted from them.”* Notably, the mental health support workers observed a reduction in depression/anxiety symptoms during the sessions. For example, participants who were known to frequently experience panic attacks or high levels of anxiety in normal group situations did not appear to suffer to the same extent when taking part in the wetland NBI. This was illustrated by the following statement:

*“It became clear quite quickly to me, how much they (the participants) managed to better manage their conditions during the sessions. They were able to switch their mind, their thoughts and their thinking processes from their issues into a much more positive outlook.”*

#### 3.2.2. Contribution to Long-Term Symptom Management

Both mental health support workers believed the wetland NBI provided participants with something to look forward to, which participants would use to better manage periods of particularly severe depression or anxiety, reminding themselves of a positive experience. The wetland NBI was described as an experience that contributed to the recovery process, one on which participants could build on long after the intervention had ended. This was illustrated by one of the mental health support workers who reflected on how participants would frequently refer to their experience on the wetland NBI to manage their ongoing symptoms in community mental wellbeing service one-to-one sessions:

*“I would categorically and unreservedly say it has been of great benefit to those individuals taking part. It is mood enhancing, it is an increase in their self-esteem. It is an important building block for the development of these individuals.”*

*“Many of my clients* (the participants) *still talk about their time at the wetland, which was now several months ago. In our one-to-one sessions they often refer to it as an example of where they achieved something or where they have taken part in something that has really helped.”*

### 3.3. Additional Associated Wetland NBI Outcomes

#### 3.3.1. Reducing Social Isolation

In addition to the effects on mental health, participants articulated several additional outcomes. Many described how being part of the wetland NBI had developed their confidence, particularly in regard to their ability to interact socially with other participants: *“I feel more relaxed and happy to talk to people more. Sometimes I can’t do that, but here I find it ok.”* This led to reduced feelings of social isolation, with a general consensus that participants had created meaningful and lasting relationships with other participants that would extend past the six-week intervention:

*“Being here I have met some wonderful people. I don’t feel so alone. I think that is really important for people like us. You do need to get out and about, but not just with family members, with other people that understand you. I think a lot of us probably feel like we don’t really fit in. I think here we just understand each other. I think we will definitely try to come back here together. I feel like I have made some great friends definitely.”*

#### 3.3.2. Increasing Confidence to be in Nature

Several participants also reflected on how they believed the wetland NBI had increased their confidence to be in nature. One participant said:

*“I wouldn’t have come somewhere like this by myself. I would have done in the past, but now my anxiety stops me. But now I have been and because you all [wetland site staff] supported me I think I would feel better about coming again. Maybe not by myself but with a friend. I think I have done a lot more because of the support from everyone than I would normally.”*

#### 3.3.3. Improving Physical Health Management

Physical benefits were also acknowledged by participants, with comments such as: *“I definitely think I feel healthier now, like physically.”* This was especially important to several participants who stated that they rarely left their homes outside of attending the wetland NBI. For these participants, the sessions made exercising easier, for example:

*“I don’t leave the house. I don’t get no real exercise. Coming here though you don’t even notice you’re doing the exercise. You’re walking around and it is good for you, but it’s not mandated. It’s enjoyable.”*

Finally, the wetland NBI was seen as a tool to help participants manage the emotional effects of pre-existing physical health conditions. One participant described this as:

*“It’s just an escape. I’m waking up with a bleeding nose every morning. I have no idea what’s happening physically in my head, let alone mentally. So to come here, I just don’t think about it. Yeah, you can literally just come here and stand by the river and listen – it’s like mindfulness. It’s that you can just focus on nothing, only on what’s around you. It’s a break from reality. A two-hour holiday.”*

### 3.4. The Role of the Natural Environment in Facilitating Wetland NBI Outcomes

There was a reoccurring theme about the value of being in an outdoor natural setting, rather than indoors. Participants shared how they felt spending time within the wetland site made them feel *“less cooped up”*. One participant put this in the context of an art therapy course they had taken before: *“It’s a lot better outdoors. I like art, like I really love art, but being outdoors is definitely for me... it’s better.”* Comments were made about the perceived peacefulness of the wetland, in addition to: *“Calmness, with no hustle and bustle like you’d find in a town centre.”* The mental health support workers also identified the *“peace and quiet”* of the wetland as a key factor contributing to the benefits experienced by participants.

Many of the participants felt being within the wetland site had provided them with a feeling of being connected to something bigger than themselves, which helped distract them from stressful life events and symptoms associated with poor mental health. For instance, participants explained this in the context of indoor health promoting interventions they had previously been part of:

*“When we go to other projects, I am just switching four walls for another four walls. This is something outside, it’s something bigger than myself. It’s something to be connected to.”*

*“There are just so many healthy distractions that you can forget about the pain in your body and your head a little bit, or worries that you have had going on in your head for hours on end.”*

Participants had difficulty identifying the relative contribution that different aspects of the natural environment played in delivering the wetland NBI outcomes they described. They did, however, frequently comment on the wildlife present at the site. Quotes included: *“When you come here, you are so engrossed in the animals and the birds all your troubles, they just disappear”* and *“the ducks and the geese make it for me. It can be so hilarious when you drop some food and all of a sudden you look behind and there are all these ducks behind you.”* The species richness supported by the wetland was also noted: *“I think walking in the wildflower meadow was fabulous for me. All that beautiful variety of plants and flowers, lots of different colours.”* Participants also described how being around water enhanced their experience: “*Water. I just love water. It is open. It is like there is nobody there.”* and *“I think just spending an hour out there on the water* [is] *relaxing. It was soothing being around the water.”* Several participants commented on the large size of the site noting that it was important as it gave them opportunities to: *“See something new each week”,* but also contributed to a sense of being *“surrounded by nature and open space.”* While the mental health support workers could not identify specific features of the wetland environment that were important, they described how they felt the ability to be *“distracted and engaged with the variety of stimuli within nature, which engaged all one’s senses”* was important in delivering the benefits participants observed.

### 3.5. The Role of the Wetland NBI Design in Delivering Outcomes

#### 3.5.1. Provision of Transportation

The logistics of getting to/from the wetland were critical to participants. There was unanimous agreement that travelling as a group via minibus relieved a lot of anxiety many would experience when going somewhere new. There was also consensus that without the minibus they would not have been able to take part in the wetland NBI, either because they did not have access to a vehicle or due to a lack of feasible public transport options. This difficulty was illustrated by one participant: *“There is an issue getting to places like this. I mean there are no buses and I am not currently able to drive. So as much as I would love to come here every day I can’t. That’s why the minibus for this was so important. It really made a difference.”*

#### 3.5.2. Engagement of NBI Staff

The importance of staff involvement was also highlighted. Many participants found that the wetland site staff were needed to help them engage with nature, through being able to get questions answered. Moreover, the enthusiasm of staff was perceived as essential: *“Your guys’* [wetland site staff] *enthusiasm has been pretty infectious. It has been really good listening and learning stuff from you and it’s been really fun”* and *“If you came here by yourself, you wouldn’t have someone to tell you what you’re looking at. It wouldn’t be as good.”*

#### 3.5.3. Session Content

While participants on the intervention did not explicitly compare the activities that took place over the six-week period, many explained sessions that involved a single activity in one part of the site were preferred. Participants felt this meant they could take in their surroundings and relax more easily, rather than feeling like they had to *“get somewhere”*. For example: *“When we went canoeing, we were there for the whole session, which was great. Some of the others were hard because we needed to go to lots of different spots to see things. It could make me feel rushed.”* Several participants discussed how longer sessions may help reduce this pressure, suggesting that it would be better to spend the whole day at the wetland. In comparison, the mental health support workers indicated that the wetland NBI should comprise a greater number of sessions across the intervention, rather than the same number but longer sessions. Indeed, eight to twelve sessions were suggested as optimal to make the experience an *“ongoing intervention that continues throughout the year.”*

#### 3.5.4. Group Dynamics

The group format of the wetland NBI was discussed, and was believed to have both a positive and negative influence on the outcomes experienced. The participants believed that being able to interact with others was important, particularly for those participants who rarely left their homes and did not have other opportunities to interact socially. It also increased participant’s confidence to be out in nature, making them feel safe: *“I wouldn’t want to go around by myself as I wouldn’t feel safe.”* However, it was noted that there were differing abilities or preferences within each group. For instance, one participant said: *“Some people walk too fast for me, because I always have to moan at them because they are walking too fast. I always have to ask for them to slow down.”* No additional aspects of the wetland NBI design were seen to be negative by participants.

## 4. Discussion

The burden of mental illness is growing, posing a significant challenge for healthcare providers [1]. Finding cost-effective and sustainable solutions to treat poor mental health is, therefore, a global priority. Nature-based health interventions (NBIs) may represent a step change in how poor mental health is managed, providing an effective, low-cost treatment option with minimal adverse side effects [28]. Despite the growing number of NBIs available, evaluations of their effectiveness are rare and have thus far primarily concentrated on NBIs taking place in green spaces. For the first time, we demonstrate that wetland NBIs can contribute to the treatment of individuals diagnosed with anxiety and/or depression.

### 4.1. Wetland NBI Outcomes

The positive impact of the wetland NBI was illustrated by significant changes across all of the measured mental health indicators. This effect was further supported by participants, with confirmatory observations made by the mental health support workers, who consistently described how the wetland NBI had improved their mental health, facilitated relaxation and enhanced positive emotions. Both relaxation and positive emotions have been associated with multiple wellbeing benefits. For example, relaxation is positively associated with the promotion of physical and mental health [43,44,45,46]. Likewise, the presence of positive emotions is linked to good health more generally, including increasing longevity [47,48] and the likelihood of engaging in future health promoting behaviours [49]. While the primary aim of the wetland NBI was to improve mental health, additional physical health and reduced social isolation benefits were also apparent. NBIs have been praised for their ability to deliver multiple positive health outcomes at once [28,50,51]. With this in mind, if scaled up, NBIs represent an effective therapy option for the treatment and prevention of numerous non-communicable diseases [28], making them an attractive solution to both healthcare providers and policy makers. Our findings provide additional support to the early calls to bring the benefits of nature interaction to healthcare [52,53].

Despite blue space being associated with a range of positive mental health outcomes, the majority of research to date has focused narrowly on coastal blue spaces (e.g., [54,55,56,57]). The value of inland wetlands for wellbeing is comparatively understudied. Different types of natural environment vary in their ability to promote wellbeing (e.g., [6,14,58,59,60]) and so it is important to discern whether, and to what extent, wetlands can also promote good mental health. For the participants in this study, spending time within a wetland environment was associated with reductions in perceived stress, a finding that is comparable to recently published work [21]. We expand this evidence-base by demonstrating wetlands additionally play a role in reducing anxiety and negative affect, while increasing mental wellbeing and positive affect. This illustrates the wellbeing benefits of wetlands are broader than simply stress reduction, with outcomes that are analogous to literature on coastal blue space and the more extensively studied green space (e.g., [61]). Wetlands therefore represent a valuable ecosystem for the promotion of mental health that, to date, has been largely overlooked.

### 4.2. Explanatory Pathways Underpinning the Outcomes

Although the participants lived in the surrounding areas, the NBI was, for many, their first visit to the wetland site and offered an escape from day-to-day life. The opportunity to spend time within the wetland site not only allowed for a physical shift from participant’s normal environments, but also a psychological one. This feeling of escape can play an important role in the relationship between nature and mental health, through alleviating oppression and emotional pain [62]. Indeed, participants in this study noted that ‘being away’ was related to the reduction of symptoms associated with their anxiety and/or depression.

The concept of ‘being away’ is related to Attention Restoration Theory (ART) [63]. ART argues that natural environments can provide cognitive benefits through restoration of the capacity to focus or direct attention [64,65], by providing features that invoke involuntary attention [66] or ‘soft fascination’ [20]. Additional proposed components of a restorative environment include ‘extent’ (the impression of providing enough to see and experience to be sufficiently engaging) and ‘compatibility’ (the setting aligns with one’s purposes and inclinations) [20]. Much of what is within a wetland setting, such as water and wildlife, can be considered inherently fascinating [63]. This is exemplified by participants making reference to the distraction that the wetland flora and fauna provided from their wider problems. In particular, they mention how it offered respite from worry and other cognitive-related symptoms of anxiety/depression. Extent was highlighted by described feelings of being encompassed by nature in a large wetland and opportunity for continued exploration. Together, this indicates that wetlands act as a restorative environment, aligning with ART, presenting a ‘fit’ between what the setting offers and the needs of individuals, which acted as a pathway for the provision of positive mental health effects [19]. These attributes should be considered when identifying environments to deliver future NBIs; ensuring the criteria of restorative environments are met can maximise the potential for good mental health outcomes.

An additional pathway that could partially explain the positive outcomes observed in this wetland NBI is the group-based social dynamic. Hartig et al. posit that the social dimension may act as a mediator between contact with nature and health through fostering social cohesion and providing a sense of community and security [67]. There is growing qualitative evidence that the social aspects of programmes that take place in nature play an important role in delivering positive mental health outcomes (e.g., [61,66]). It is likely that multiple pathways are engaged simultaneously to explain the positive outcomes of this NBI [68]. This additive or potentially synergistic relationship needs to be further understood to maximise the effectiveness of future NBIs.

### 4.3. Importance of NBI Design

As part of this pilot, we wanted to address calls for evidence on the design of NBIs and aspects that contributed to the success or failure of interventions [28]. The NBI offered a structure to facilitate nature engagement and benefit from the restorative qualities of wetlands. However, it is clear from this work that there were numerous additional features of the wetland NBI that participants believed made it successful. The inaccessibility of natural environments is often referred to as a barrier to experiencing the benefits of nature [69]. The provision of transport to/from the wetland reduced this challenge, which is of particular importance to people experiencing anxiety and/or depression. It avoided the anxiety several participants felt they would have experienced if trying to arrange their own travel to the wetland site, and for many who did not have access to a vehicle was essential to be able to participate in the NBI. The involvement and environmental knowledge of the wetland site staff were also seen as integral to the success of the NBI. Participants appreciated the wetland site staff pointing out species during activities. It has been proposed that perceived biodiversity, rather than actual biodiversity, contributes most significantly to self-reported wellbeing [25,70]. Therefore, drawing attention to the diversity of species around participants may enhance levels of perceived biodiversity and, consequently, promote the positive outcomes of the wetland NBI. Irrespective of the mechanisms behind this relationship, the staff engagement was clearly an important component of the NBI and should be considered in the design of future interventions.

The group component of the NBI was associated with both positive and negative comments, with the latter relating to differences in abilities among participants. Previous research has highlighted the moderating role that the social context can play in fostering wellbeing when, for example, an individual is on their own or with others (e.g., [71]), and whether the group is structured or unstructured (e.g., [72]). We recommend designers of existing and future NBIs consider how to manage the group dynamic for the greatest benefit for all participants. One option would be to divide groups on the basis of characteristics such as speed of walking. This approach could reduce the perceptions of being rushed, while ensuring the number of activities provided each week allows for sufficient stimulation and variety.

### 4.4. Valuing the Impact of the Wetland NBI

Monetary valuation of NBIs for the treatment of specific conditions is rare, focusing on costs saved to healthcare services, with examples predominantly limited to grey literature (e.g., [73]). While the sample size of this pilot is small, we provide an indicative estimate of the value of our NBI outcomes to facilitate comparisons with other wellbeing interventions. To do this, we draw on the Wellbeing Valuation approach that is commonly adopted by the UK government and non-governmental [74,75], which uses subjective measures to drive marginal rates of substitution between a non-market good and household income [75]. The Mental Health Social Value Calculator (https://www.hact.org.uk/mental-health-social-value-calculator) applies the Wellbeing Valuation approach to WEMWBS data. Using participants’ WEMWBS scores before and after the wetland NBI, the average wellbeing effect was valued at £4848. Previously, this approach has been applied to interventions such as arts engagement or sports participation, which have been valued at £1084 and £1127 per person respectively [76]. While this may suggest the wetland NBI benefits are particularly valuable, there was substantial variation across our small sample of participants (range: £0–£16,314) that must be considered. Moreover, this figure does not include the cost of delivering the wetland NBI. While much of the delivery came out of existing staff capacity, this still has an economic cost to organisations. In addition, costs for transport incur substantial expenses, which would need to be incorporated in future full cost-benefit analyses. Additionally, given the Wellbeing Valuation approach relies on a single mental health indicator, it does not capture the range of benefits derived by participants. As such, the economic valuation of the NBI may undervalue outcomes. While this method may be simple for organisation to use, it can only be relied on to provide an indication the NBI economic value. It does not replace the need to perform full cost-benefit analysis of NBIs. More work is needed to evaluate the usefulness of existing approaches to cost-benefit analysis to determine whether they are appropriate to apply to NBIs. Ideally, new methods would be developed that more effectively incorporate the multifaceted outcomes of NBIs, not least because we see from this pilot that the benefits of partaking in NBIs are multifaceted and often complex.

### 4.5. Limitations of the Wetland NBI Pilot

There are several limitations associated with this pilot that should be considered when interpreting the findings and conducting further research. One limitation of our study was the small sample size. This was constrained by the requirements associated with the study population, which was determined by the guidance of the community mental wellbeing service. It was necessary to ensure groups were small enough to not detract from the experience for participants who experienced anxiety in large groups. An additional limitation was that participants were relatively homogenous in sociodemographic characteristics, and did not reflect the diversity of the wider population experiencing anxiety and/or depression. Nevertheless, the sample generally mirrored the community mental wellbeing service membership. Finally, this was a pilot study and consequently was only conducted at one wetland site with a specific single design for the NBI. Future work could aim to replicate this study to increase the sample size and evaluate alternative NBI designs, within a controlled framework.

## 5. Conclusions

We demonstrate wetland NBIs can be valuable for the treatment of individuals experiencing anxiety and/or depression by improving mental health. This paper addressed calls for evidence of the effectiveness of NBIs and how the natural environment and intervention design might contribute to wellbeing outcomes. We propose that the relationship between the natural environment and the positive mental health outcomes can be explained by existing theories, including ART. Akin to other blue spaces, results indicate the ability for wetlands to act as restorative environments and promote health and wellbeing, mediating the outcomes of the NBI. We highlight the contribution of transportation, group dynamics and staff input to the success of NBIs, however the ability for organisations to incorporate each of these elements into intervention design will be limited by the availability of funding.

## Figures and Tables

**Table 1:** Sociodemographic background of participants who took part in the wetland nature-based health intervention (NBI) (n = 16) in comparison to the wider community mental wellbeing service membership (n = 851).

Sociodemographic Background	Wetland NBI		Community Mental Wellbeing Service	
	N	%	N	%
Gender	n = 16		n = 851	
Male	8	50	406	48
Female	8	50	445	52
Age	n = 16		n = 851	
18–29	3	19	190	22
30–49	5	31	341	40
50–64	3	19	300	35
65–84	5	31	20	3
Ethnicity	n = 16		n = 508	
White—British	13	81	477	94
White—Other	3	19	17	3
Any Black background	0	0	6	1
Any Asian background	0	0	4	1
Other ethnic background	0	0	4	1
Employment status	n = 16		n = 209	
Currently unemployed	10	63	163	78
Retired	5	31	13	6
Student	1	6	1	1
Employed (Full-time)	0	0	12	6
Employed (Part-time)	0	0	14	7
Voluntary work	0	0	5	2
Home environment	n = 16		Not asked of wider membership	
Urban	8	50		
Rural	8	50		

**Table 2:** Indicators of mental health Wilcoxon signed rank test, measured before and after the six-week wetland nature-based health intervention (n = 16).

Measure ^a^	Pre-Intervention Mean (± SE)	Post-Intervention Mean (± SE)	*z*	*p*
WEMWBS	37 (± 2.79)	41 (± 4.31)	−2.60	0.009
GAD-7	13.27 (± 1.54)	10.28 (± 1.46)	−3.02	0.002
PSS	24.31 (± 2.54)	22.35 (± 1.47)	−2.04	0.041
PANAS (positive)	26 (± 2.64)	30.57 (± 3.08)	−2.49	0.012
PANAS (negative)	28.63 (± 2.99)	27.71 (± 3.66)	−2.24	0.025

^a^ Warwick and Edinburgh Mental Wellbeing Scale (WEMWBS); Generalised Anxiety Disorder Scale (GAD-7); Perceived Stress Scale (PSS); Positive and Negative Affect Schedule (PANAS).
